# Awareness, understanding, and interest in personalized medicine: A cross-sectional survey study of college students

**DOI:** 10.1371/journal.pone.0280832

**Published:** 2023-01-25

**Authors:** Yingke Xu, Qing Wu

**Affiliations:** 1 Nevada Institute of Personalized Medicine, College of Sciences, University of Nevada, Las Vegas, Las Vegas, Nevada, United States of America; 2 Department of Epidemiology and Biostatistics, School of Public Health, University of Nevada, Las Vegas, Las Vegas, Nevada, United States of America; 3 Department of Biomedical Informatics, College of Medicine, The Ohio State University, Columbus, OH, United States of America; Al-Jouf University College of Medicine, SAUDI ARABIA

## Abstract

**Introduction:**

Personalized Medicine (PM) holds great potential in healthcare. A few existing surveys have investigated awareness, understanding, and interest regarding PM in the general public; however, studies investigating college students’ opinions about PM are lacking. This study aimed to evaluate the college student’s awareness, understanding, and interest in PM, and their opinion was also analyzed by their gender and major.

**Methods:**

The study samples were undergraduate students enrolled at the University of Nevada, Las Vegas (UNLV). A web-based survey with 42 questions was emailed to all UNLV undergraduate students. Overall survey results were analyzed by gender and each student’s major. A chi-square test evaluated the significant association between responses to questions with regard to gender or major.

**Results:**

Among the participants, 1225 students completed the survey. This survey found that most college students had a neutral attitude to PM and were not entirely familiar with this field. For example, most students (57.6%) had a “neutral” attitude toward PM. In addition, 77.6% of students never received any personal genetic testing. More than 80% of students thought “interests” was the most important factor in using PM, and 50% of respondents chose “somewhat likely” to the recommendation about PM from the doctor. Also of importance was the finding that a significant association between the most important factor of using PM and gender was observed (p = 0.04), and the associations between a student’s major affected his or her reaction to PM, how well informed she or he was about PM, his or her attitude toward a doctor’s recommendation about using PM were all significant (all participant’s p<0.004).

**Conclusion:**

UNLV undergraduate students had a neutral attitude to PM and were not entirely familiar with this field.

## Introduction

Personalized Medicine (PM), an approach that tailors healthcare to individual genetic profiles [1], promises more customized and efficient diagnosis and treatment when utilized in clinical practice. With recent technology developments such as DNA sequencing, health professionals now realize the heterogeneity of disease processes [1]. The underlying heterogeneity of diseases indicates that personalized strategies for preventing, monitoring, and treating diseases that are based on an individual’s unique biochemical, physiological, environmental exposure, and behavioral profile are desperately needed. The cost of sequencing technology has decreased dramatically, thus allowing easy public access [2]. In 2015, 23andMe, a genetic testing company, genotyped over 1 million customers worldwide to help people better understand their health [3], suggesting that the novel technology has become more popular in recent years. Advances in the development of genetic tests and therapies provide the potential to create the unprecedented ability for detection, prevention, and treatment of diseases [4]. Over the last few decades, therapy approaches based on genetic variants, and specific biomarkers have increased. For example, the expression of specific oncogene products based on translocations of specific genes and the presence of specific mutations have been used as so-called molecular targets, such as erlotinib for EGFR mutations [5] alectinib for ALK fusion genes [6] showed high antitumor efficacy. However, the increased utilization of PM raises concerns about the regulation and clinical application to people. The World Economic Health Forum’s Precision Medicine Program has advocated supporting the development of policy frameworks and governance protocols to realize the societal benefits and mitigate the risks from PM [7]. The public is one of the most critical stakeholders in PM, but there have been limited studies focusing on the public’s awareness of and opinion about this field. Understanding the public’s perceptions about PM is vital because the public is the primary beneficiary and funder [8]. Furthermore, knowing the perceptions among college students is significant as well. Given more and more young people are choosing a career in the healthcare industry [9], an accurate survey of college students’ knowledge and understanding of the field will help schools offer related curricula to enhance their competence and ultimately boost their employability in the future job market. Two related surveys showed that Americans are not familiar with PM [10, 11]; however, these surveys were conducted either among the general population or medical school students. As well, few studies focus on general college student populations, and thus the awareness and attitude toward PM among college students remain unclear.

This study aimed to survey students at the University of Nevada, Las Vegas (UNLV) in order to assess their awareness and opinions of PM. Specifically, this study examined any difference in students’ opinions about PM, especially in the areas of gender or major. Additionally, considering the difference between men’s and women’s perception of disease symptoms and treatment [12–16], this study also aimed to evaluate whether awareness, understanding, and interest in PM differed by gender.

## Materials and methods

### Study design and administration

The cross-sectional survey was conducted at UNLV from 06/2016 to 04/2017. The survey sample was from 29,702 undergraduate students of UNLV who were enrolled during the fall semester of 2016. A list of university-associated email addresses was obtained from the UNLV Office of the Registrar. The web-based survey form was emailed to all students on the list. Each undergraduate student received a unique link and an invitation to participate in the study and questionnaire through email. The system sent three follow-up emails to remind all non-responsive undergraduate students to fill out the survey to collect as much data as possible. Each participant could use a back button to check their selections. The confidential Research Electronic Data Capture (REDCap) web tool [17], hosted at the UNLV Nevada Supercomputing Institute, was employed to administer the survey and collect all responses. REDCap is an electronic data capture system developed by Vanderbilt University and can be used to design electronic case report forms and edit checking programs without having professional knowledge of the software [18]. The REDCap’s Participant List option allows researchers to send a customized email to anyone in the list and track who responds to your survey [7, 17]. Participant information was de-identified by the REDCap before the data was exported for analysis. The participants were informed of the study’s purpose prior to their participation; they had the option to quit at any time. The respondents also provided digital consent for their information to be used for this study. The UNLV Institutional Review Board approved this study under protocol #888139.

This survey included 42 well-structured questions (see Appendix) adapted from a questionnaire created by the Personalized Medicine Coalition [11]. These questions focused on the following sections: reaction to PM, awareness of PM, what kind of personal genetic testing the respondent ever used, and the crucial deciding factor in pursuing personal genome sequencing. Cronbach’s alpha was used to assess the tool’s reliability, and the value was 0.61. In addition, demographic information from respondents was collected to better understand the awareness and attitude among students by gender and major. UNLV faculty members with a background in epidemiology and personalized medicine survey research approved all questions. The study was carried out following the Checklist for Reporting Results of Internet E-Surveys (CHERRIES) guidelines [19].

### Statistical analysis

Cochran’s formula (n = z^2^pq/e^2^) was used for the sample size calculation. In this formula, z is desired confidence interval, which was set as 95% in this study; p is the proportion, and q = 1-p; p = 0.5 was set in this study to generate the most conservative sample size [20]; e is the desired level of precision, which was set at 5%; the estimated sample size was 385. For each question, the percentage of participants who selected responses was calculated. The responses of participants were assessed by gender as well as major. A chi-square test was utilized to evaluate the significant association between responses to questions and gender or major. Ordinal logistic regression was used to identify factors associated with the participants’ reactions and if they were well-informed about personalized medicine. Students with a DO NOT RELEASE flag were not included for analysis. The significance level was set at p  =  .05, and all analyses were conducted using SAS version 9.4 (SAS Institute, Cary, NC, USA).

## Results

A total of 1288 UNLV undergraduate students participated in this survey, and 1225 completed the questionnaire. The characteristics of these respondents are shown in Table 1. A total of 790 (64.49%) participants were women; 509 (41.55%) were Caucasian, 299 were Asian (24.41%), and 268 were Hispanic (21.88%); 408 participants were senior students (33.31%), and 338 (27.59%) were junior students; 467 (38.12%) were science majors, and 288 (23.51%) were art majors.

**Table 1:** Respondent demographic characteristics.

Characteristics	No. (%), N = 1225
Sex	
Men	435 (35.51)
Women	790 (64.49)
Race	
Caucasian	509 (41.55)
African American	82 (6.69)
Hispanic	268 (21.88)
American Indian/ Alaska Native	15 (1.22)
Asian	299 (24.41)
Don’t know/refused	52 (4.24)
Class standing	
Freshman	239 (19.51)
Sophomore	240 (19.59)
Junior	338 (27.59)
Senior	408 (33.31)
Major	
Bachelor of Arts	288 (23.51)
Bachelor of Fine Arts	75 (6.12)
Bachelor of Landscape Architecture	5 (0.41)
Bachelor of Music	12 (0.98)
Bachelor of Science	467 (38.12)
Bachelor of Science in Business Administration	153 (12.49)
Bachelor of Science in Engineering	134 (10.94)
Bachelor of Science in Public Administration	37 (3.02)
Bachelor of Social Work	54 (4.41)

**Fig 1:**
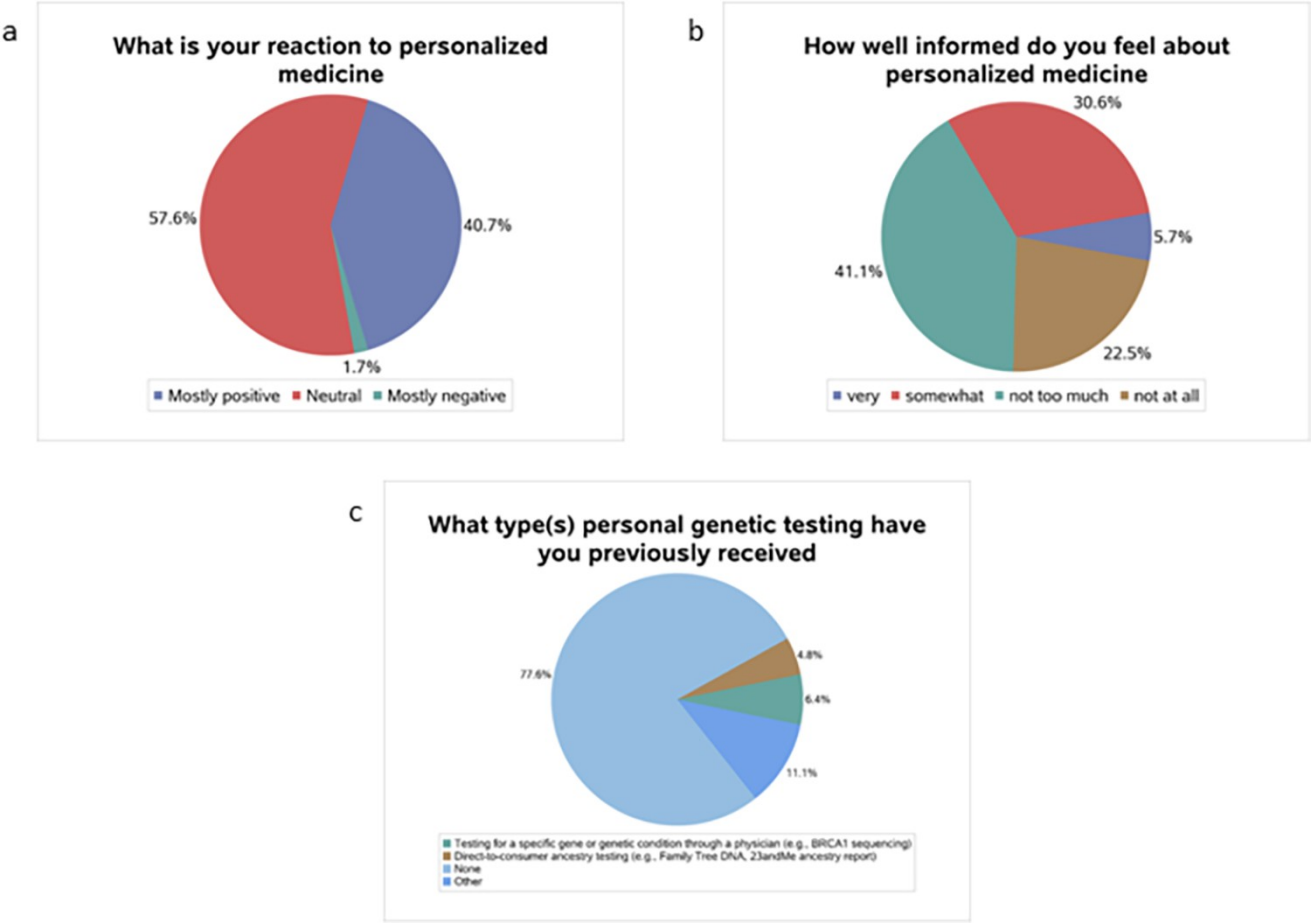
Respondent’s awareness of personalized medicine. (**a**) What is the reaction of participants to the personalized medicine; (**b**) how well informed did participants feel about personalized medicine among participants; (**c**) what type(s) of personal genetic testing have participants previously received.

Fig 1 shows the awareness of PM among all participants. The majority (57.6%) of students have a “neutral” reaction to PM (Fig 1A), and 41.1% of students thought they were “not too much” informed about PM (Fig 1B), while 77.6% of respondents “had never received any genetic testing” (Fig 1C). Table 2 shows the respondents’ awareness of PM by gender. For the question regarding the reaction to PM description, the majority of men (58.62%) and women (56.96%) had a “neutral” reaction. Similar to the overall results, around 40% of men and women thought they were “not too much” informed about PM. Furthermore, 78.62% of male students and 64.49% of female students never received genetic testing. However, no significant association between students’ awareness of PM and gender was observed (all p ≥0.11).

**Table 2:** Respondent awareness and interest in personalized medicine by gender.

	Men (N = 435)	Women (N = 790)	P-value
Awareness			
Reaction to the description of personalized medicine			
Most positive	172 (39.54)	327 (41.39)	0.81
Neural	255 (58.62)	450 (56.96)	
Most negative	8 (1.84)	13 (1.65)	
Well informed do you feel about personalized medicine			
Very	30 (6.9)	40 (5.06)	0.53
somewhat	136 (31.26)	239 (30.25)	
Not too much	176 (40.46)	328 (41.52)	
Not at all	93 (21.38)	183 (23.16)	
The type of personal genetic testing that you have received			
Carrier screening through a physician	14 (3.22)	33 (4.18)	0.11
Prenatal diagnosis through a physician	10 (2.30)	35 (4.43)	
Testing for a specific gene or genetic condition through a physician	24 (5.52)	55 (6.96)	
Direct-to-consumer ancestry testing	24 (5.52)	35 (4.43)	
Direct-to-consumer health testing	14 (3.22)	19 (2.41)	
Other	7 (1.61)	4 (0.51)	
None	342 (78.62)	609 (64.49)	
**Interest & attitude**			
The most important in your decision to pursue personal genome sequencing			
Interests	360 (82.76)	663 (83.92)	0.04
Desire to participate in research	35 (8.05)	36 (4.56)	
The impact of family members	33 (7.59)	81 (10.25)	
Other	7 (1.61)	10 (1.27)	
What is your attitude if your doctor recommended a diagnostic test for use in developing a personalized prevention or treatment plan			
Very likely	180 (41.38)	319 (40.38)	0.77
Somewhat likely	211(48.51)	401 (50.76)	
Not too likely	34 (7.82)	57 (7.22)	
Not at all likely	10 (2.30)	13 (1.65)	
The government should put more effort into regulating personal genome sequencing			
Strongly disagree	35 (8.05)	67 (8.48)	0.37
Somewhat disagree	59 (13.56)	86 (10.89)	
Neither agree nor disagree	194 (44.60)	343 (43.42)	
Somewhat agree	85 (19.54)	188 (23.80)	
Strongly agree	62 (14.25)	106 (13.42)	

**Fig 2:**
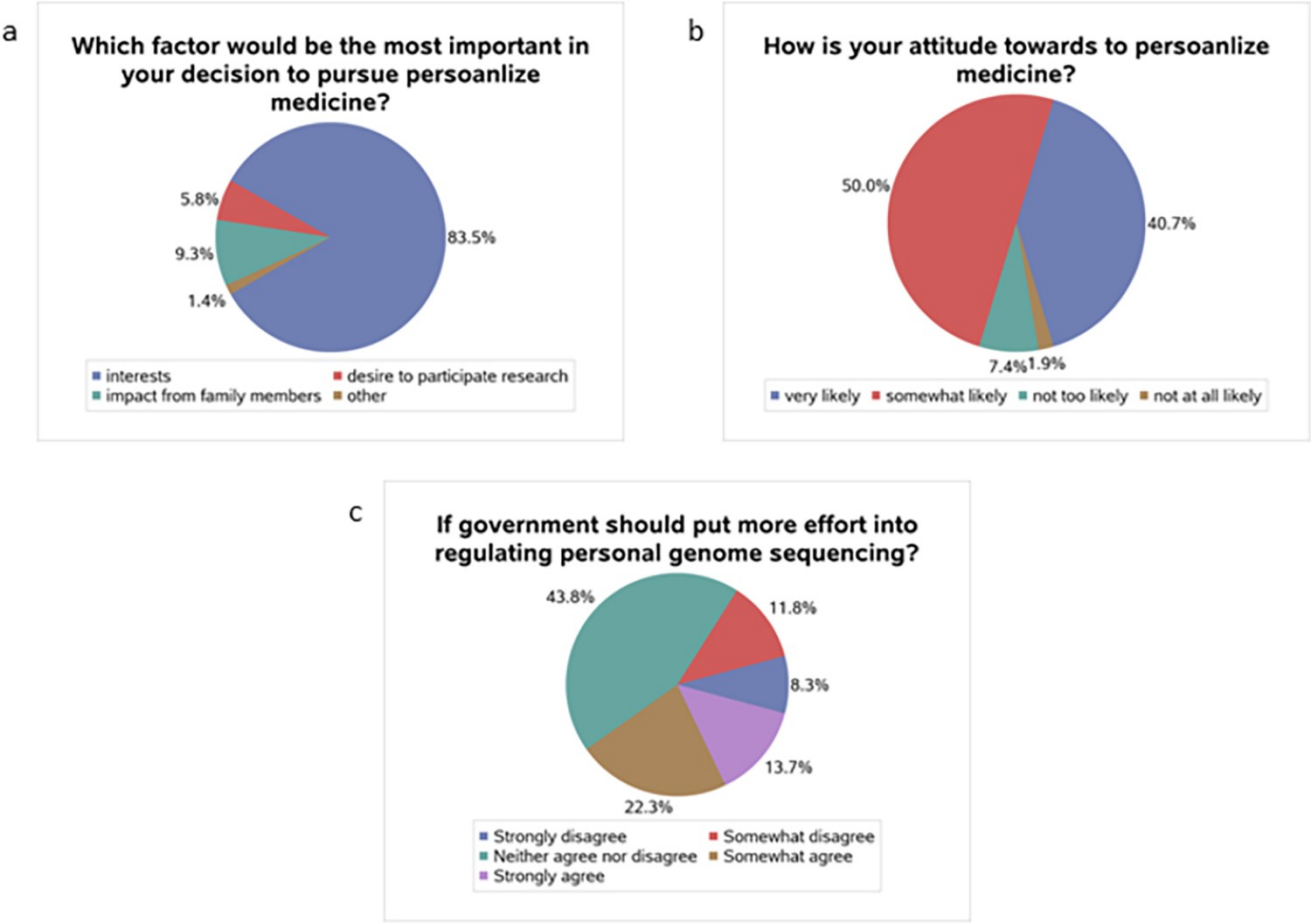
Respondents’ interest and attitude toward personalized medicine. (**a**) which factor would be the most important in participants’ decision to pursue personalized medicine; (**b**) How is participants’ attitude toward personalized medicine; (**c**) if governments should put more effort into regulating personal genome sequencing.

Fig 2 shows the interest and attitude of PM among all respondents. For 83.5% of participants, “interests” is the most important factor in PM use (Fig 2A). 50.0% of students had a “somewhat likely” attitude to a recommendation about PM from the doctor (Fig 2B), while 43.8% of participants were “neither agree nor disagree” about if the government should put more effort into regulating personal genome sequencing (Fig 2C). The interest in and attitude toward PM among students by gender are shown in Table 2. For the majority of men (82.76%) and women (83.92%), “interests” was the essential factor in pursuing personal genome sequencing. Also, around 8.05% of male students thought the “desire to participate in research” was the most important factor in the use of personal genome sequencing. In comparison, 10.25% of females thought the “impact of family members” was the most important factor. A significant association appeared between the most important factor of using personal genome sequencing and gender (p = 0.04). Regarding doctors’ recommendation for using PM, around 49% of men and women have a “somewhat likely” attitude. For both genders, around 44% of participants had a neutral attitude regarding if the government should put more effort into regulating personal genome sequencing.

The awareness of students by major was also assessed (Table 3). For students majoring in science, the majority (50.75%) have a “most positive” reaction to the description of PM, while 63.32% of students majoring in non-science have a “neutral” reaction to it. For students majoring in science, 38.97% and 37.47% thought they were “not too much” and “somewhat” well informed about PM, respectively. For students with non-science majors, 42.48% and 26.91% of students thought they were “not too much” and “not at all” well informed about PM. Further, there were significant associations between students’ majors, their reaction to PM, and how well-informed PM (both p<0.0001). Table 3 shows the interest and attitude of PM among respondents by major. For students in science and non-science majors, more than 80% of students chose “interests” as the most important factor in pursuing personal genome sequencing. Notably, most students with science majors thought they were “very likely” to use PM if a doctor recommended it for a diagnostic test. However, for students with non-science majors, most of them responded “somewhat likely” to the same question. A significant association was observed between the participants’ attitude to doctors’ recommendation of using PM and their major (p = 0.0004).

**Table 3:** Respondent awareness and interest in personalized medicine by major.

	Bachelor of Science (N = 467)	Non-Bachelor of science (N = 758)	p-value
**Awareness**			
Reaction to the description of personalized medicine			
Most positive	237 (50.75)	262(34.56)	<0.0001
Neural	225 (48.18)	480 (63.32)	
Most negative	5 (1.07)	16 (2.11)	
Well informed do you feel about personalized medicine			
Very	38 (8.14)	32 (4.22)	<0.0001
somewhat	175 (37.47)	200 (26.39)	
Not too much	182 (38.97)	322 (42.48)	
Not at all	72 (15.42)	204 (26.91)	
The type of personal genetic testing that you have received			
Carrier screening through a physician	12(2.57)	35 (4.62)	0.32
Prenatal diagnosis through a physician	15 (3.21)	30 (3.96)	
Testing for a specific gene or genetic condition through a physician	33 (7.07)	46 (6.07)	
Direct-to-consumer ancestry testing	28 (6.00)	31(4.09)	
Direct-to-consumer health testing	14 (3.00)	19(2.51)	
Other	3 (0.64)	8(1.06)	
None	362 (77.52)	589 (77.70)	
**Interest & attitude**			
The most important in your decision to pursue personal genome sequencing			
Interests	400 (85.65)	623 (82.19)	0.31
Desire to participate in research	26 (5.57)	45 (5.94)	
The impact of family members	37 (7.92)	77 (10.16)	
Other	4 (0.86)	13(1.72)	
What is your attitude if your doctor recommended a diagnostic test for use in developing a personalized prevention or treatment plan			
Very likely	219 (46.90)	280 (36.94)	0.004
Somewhat likely	213 (45.61)	399 (52.64)	
Not too likely	30 (6.42)	61 (8.05)	
Not at all likely	5 (1.07)	18 (2.37)	
The government should put more effort into regulating personal genome sequencing			
Strongly disagree	36 (7.71)	66 (8.71)	0.30
Somewhat disagree	60 (12.85)	85 (11.21)	
Neither agree nor disagree	195 (41.76)	342 (45.12)	
Somewhat agree	101 (21.63)	172 (22.69)	
Strong agree	75 (16.06)	93 (12.27)	

The results of ordinal logistic regression are shown in Table 4. The results clearly indicate that major and class standing significantly impacted the participants’ reaction and familiarity with PM. Specifically, participants who majored in science and were seniors had more positive reactions to PM (both p ≤0.002) and felt better informed about PM (both p≤0.001).

**Table 4:** Ordinal logistic regression results for participants’ reaction and well informed to personalized medicine.

Response	Predictors	β	p-value	Odds ratio (95% CI)
Reaction to personalized medicine	Gender			
	Male	Ref	Ref	Ref
	Female	0.04	0.75	1.04 (0.82, 1.32)
	Major			
	Non-science	Ref	Ref	Ref
	Science	0.68	<0.0001	1.98 (1.56, 2.50)
	Class standing			
	Freshman	Ref	Ref	Ref
	Sophomore	0.04	0.82	1.04 (0.72, 1.53)
	Junior	0.25	0.15	1.28 (0.91, 1.81)
	Senior	0.51	0.002	1.67 (1.20, 2.31)
Well informed in personalized medicine	Gender			
	Male	Ref	Ref	Ref
	Female	-0.18	0.11	0.84 (0.68, 1.04)
	Major			
	Non-science	Ref	Ref	Ref
	Science	0.69	<0.0001	1.99 (1.60, 2.47)
	Class standing			
	Freshman	Ref	Ref	Ref
	Sophomore	0.09	0.59	1.10 (0.79, 1.52)
	Junior	0.21	0.17	1.24 (0.91, 1.68)
	Senior	0.50	0.001	1.64 (1.22, 2.21)

## Discussion

This survey evaluated the awareness and attitudes toward PM among college students from UNLV. Specifically, more than half of students (57.6%) have a “neutral” attitude toward PM. 77.6% of students never received any personal genetic testing. More than 80% of students thought interest was the most important factor in using PM, and 50% of respondents chose “somewhat likely” to use after a recommendation about PM from a doctor. Also of interest, a significant association between the most important factor of using PM and gender was observed (p = 0.04), while the associations between the respondents’ reaction to PM, feeling well-informed about PM, attitude to the doctor’s recommendation about using PM, and major were significant (all p<0.004). Additionally, ordinal logistic regression results showed that participants’ major and class standing significantly impacted their reaction and familiarity with PM. In addition, a significant association appeared between the most important factor of using personal genome sequencing and gender (p = 0.04). Significant associations were observed between students’ major and their reaction to PM and between students’ major and if they felt well-informed about PM (both p<0.0001).

The results of this study correspond to prior studies about PM. In this survey, the significant association between attitude about PM and major indicates that science majors had a more positive attitude toward PM and were more familiar with this field than non-science majors. This result corresponded to a previous study, which found that medical students supported using PM but did not feel prepared for it [10]. In the current study, the different attitudes could be due to science majors having learned PM-related information during their studies, such as in biology and in genetics. However, students who were not in this major might have limited access to knowledge about it. Also, another previous study found that students with science majors outperformed non-science majors in genetic learning [21], which might also explain the attitude and feeling well informed about PM among students in the two majors. Another study conducted among healthcare professionals also confirmed that limited knowledge and negative attitudes were associated with a lack of awareness of PM [22], which also corresponded to our results. Our survey found that most students were “not too much” informed about PM. Most of them never received personal genetic sequencing. The result was consistent with a recent survey conducted in Hong Kong that undergraduates showed a high awareness of PM but insufficient genetic-related knowledge [23].

The lack of related knowledge and awareness could lead to severe obstructions in utilizing PM in practice. College students, especially science-major students, could be future healthcare workers, so addressing gaps in genomics/PM education is essential. More than 80% of respondents thought interest was the most important factor in using personal genomic sequencing, and 50% of respondents had a “somewhat likely” attitude toward a doctor’s recommendation for using PM, indicating these particular respondents might be interested in PM. Although many institutions have developed no-cost resources that provide essential scientific explanations of PM principles and technology-specific details to students, more strategies and interventions are needed to integrate PM into the college curriculum. The participants’ opinions about regulation and factors for pursuing PM were also informative, which could help the government address potential challenges and issues in implementing and using PM, thus accelerating PM development in the health care system.

### Limitations

In this study, several limitations should be considered in interpreting the findings. The first limitation is that this was an online survey, so drawing a sample based on email addresses is more challenging. Therefore, a type of non-probability sampling method, convenience sampling, was used in the current study; such a non-probability method might lead to a generalizability issue since such sampling method might not select representative samples from the population. Over-or under-representation could also be a concern [24]. Another limitation of convenience samples is that normally small numbers of underrepresented subgroups are included, resulting in insufficient power to detect subgroup differences [25]. In addition, all surveys can carry bias due to nonresponse, and internet surveys are also vulnerable to this bias [26]. Consequently, the pool of respondents may differ significantly in this aspect from the general population. In this survey, three follow-up emails were sent to remind all non-responsive undergraduate students to fill out the survey so as allow us to collect as much data as possible. Third, this study was conducted from 2016 to 2017; the results might not reflect current student awareness, understanding, and interest in PM. However, since no similar survey was conducted among college students in recent years, the current study results are still relevant and informative since they indicated that PM-related education might help college students have a more positive attitude and a better understanding of PM.

## Conclusion

This survey has important implications in that college students have a natural attitude toward PM but are not entirely familiar with this field. Notably, students who majored in science and non-science had different awareness and attitudes toward PM. As the healthcare system transitions from its traditional, one-size-fits-all approach toward a personalized medicine paradigm, it will inevitably need to prepare college students—the potential healthcare workforce and stakeholders of tomorrow——with much-needed genomic literacy.
